# A community-based pilot randomised controlled study of life skills classes for individuals with low mood and depression

**DOI:** 10.1186/s12888-015-0384-2

**Published:** 2015-02-06

**Authors:** Carrie-Anne McClay, Katrina Collins, Lynsay Matthews, Caroline Haig, Alex McConnachie, Jill Morrison, Pat Lynch, Louise Waters, Ilena Day, Grainne McAnee, Christopher Williams

**Affiliations:** Institute of Health and Wellbeing, University of Glasgow, Gartnavel Royal Hospital, Administration Building, 1055 Great Western Road Glasgow, Glasgow, G12 0XH Scotland; AWARE Defeat Depression, 56 Strand Road, Derry, BT48 7AJ Ireland; Robertson Centre for Biostatistics, Boyd Orr Building, University of Glasgow, Glasgow, G12 8QQ Scotland; General Practice & Primary Care, Institute of Health and Wellbeing, University of Glasgow, 1 Horselethill Road, G12 9LX Glasgow, Scotland; Action on Depression, 11 Alva Street, Edinburgh, EH2 4PH Scotland; Institute of Health and Wellbeing, University of Glasgow, Gartnavel Royal Hospital, 1st Floor Administration Building, 1055 Great Western Road Glasgow, Glasgow, G12 0XH Scotland

**Keywords:** Depression, Guided self-help, Cognitive behavioural therapy, Low intensity, Living life to the full classes

## Abstract

**Background:**

Cognitive behavioural therapy (CBT) is recommended for the treatment of depression and anxiety. However, access is limited. Low-intensity approaches such as guided CBT self-help (bibliotherapy) can increase access to treatment and is recommended by UK guidelines. No previous research has explored the provision of group-based guidance/support for a bibliotherapy approach for depression and anxiety in community settings. The objective was to carry out a pilot study of a group guided self-help intervention, using community based recruitment methods.

**Method:**

A randomised controlled trial comparing an 8 week CBT group guided self-help intervention to usual care. Recruitment and the delivery of the intervention were carried out in Glasgow and Derry/Londonderry in partnership with national depression charities. Fifty-three people were randomised, however we refer only to the forty-six participants who provided baseline data: 16 males and 30 females, aged 16 or over, with a PHQ-9 score of ≥ 5, were recruited from the community. The mean age of the sample was 43.7 (sd = 13) and 93.5% of participants had suffered from low mood for a year or more.

**Results:**

There was effective recruitment, randomisation, uptake and adherence with 21 Immediate Access (IA) and 25 Delayed Access Control (DAC) participants. The intervention was highly acceptable to participants attending on average 4.46 of the 8 sessions (sd 3.06), 65.2% attended more than half of all sessions. The mean satisfaction on the Client Satisfaction Questionnaire was 28 out of 32 (sd 4.8). The provisional results in the pilot suggest the intervention may improve both anxiety and depression. At three months, data collection was achieved from 74% of participants. The trial successfully provided estimates of the sample size needed for the future planned trial.

**Conclusions:**

Low-intensity group-based classes may offer an alternative method of managing depression and anxiety and warrant further research.

**Trial registration:**

Current Controlled Trials ISRCTN84893887. Registered 3 November 2011.

## Background

The National Institute for Health and Care Excellence (NICE) states that Cognitive Behavioural Therapy (CBT) should be offered to people with moderately low mood [1]. CBT has been effective in reducing depressive symptoms within primary care [2], however, it remains difficult to provide high intensity specialist CBT therapy due to the large volume of patients with depression and, as a result, waiting lists are long. An alternative is to supplement high-intensity delivery with low intensity CBT [3]. This can be successfully achieved by utilising a guided self-help approach [4,5].

In the UK, low intensity interventions are recommended for many ‘common mental health problems’ [1] as a first step in a stepped care treatment model [6] in which patients receive the least restrictive treatment available and progress is continually monitored and adapted if necessary. Treatment options range from self-help (bibliotherapy or computerised resources) with guidance via email, telephone, face to face, group interventions and individual specialist therapy. One of the aims of the stepped care model is to reduce waiting lists for individual therapy and increase the capacity and efficiency of services [6]. This approach is utilised in the Increasing Access to Psychological Treatment (IAPT) government programme across England which was introduced due to the commitment to widening access to psychological therapies for mental health problems such as depression, anxiety and eating disorders.

Usually those using guided CBT self-help for depression are recommended to receive one to one support [1]. However, although low intensity in format, this approach still utilises one to one contact by phone or face to face and can result in delays. A potentially higher capacity approach would be to deliver CBT self-help with guidance and support within a group/class based setting. Group based support means that 10–15 people can be supported in a relatively short space of time, during a 1.5 hour session for example. If support was delivered on a one to one basis, only 4 people could be supported in this time (based on 20–30 minutes per person), using up greater resources (therapist/support worker’s time, therapy rooms etc.). Therefore, class based guided self-help has the potential to increase capacity and reduce waiting times.

This approach differs significantly from the delivery of traditional CBT groups such as The Coping With Depression (CWD) Course [7] which usually have incorporated high intensity content, delivered by CBT experts. In contrast, integral to the low intensity model is that non-expert practitioners can support the use of the materials by the person with depression. The idea is that the bibliotherapy materials themselves deliver the CBT content [8]. Although some work has been done in this area with single session stand-alone confidence classes [9] for depression, and for anxiety [10], no studies, we are aware of, have used classes as a medium of guidance/support for low intensity, bibliotherapy-based CBT life skills programmes aimed at depression.

Finally, there has been debate about how best to recruit people with depression into services. It has long been known that there is a significant treatment-gap where over half of those people with diagnosable depression fail to present to health care services for support [11]. Service delivery that encourages self-referral has shown significant benefits and more accurately reflects the demographic profile of the local community [12]. The support and guidance is important and improves outcomes of bibliotherapy for depression [13] and is recommended by NICE. Supporters do not need to be mental health experts so support can be delivered in community settings and with a non-health service label [13].

We, therefore, aimed to test our ability to recruit using self-referral from community settings, to describe the population recruited, and then test the delivery of such a course, supported by group facilitators provided by two national charities. The findings will inform a future substantive RCT of the intervention [14] and aid the delivery of the course in community settings.

## Methods

### Design

This study was a multi-centre, parallel; two-arm pilot randomised controlled trial with 50:50 ratio across the two groups. The study assessed immediate access group (IA) to an 8-week community-based life skills course for depression and anxiety versus a delayed access control (DAC) group which received the intervention approximately 14 weeks after randomisation. Classes were delivered within the voluntary sector in two countries within community settings.

### Recruitment and participants

The aim was to recruit up to 50 participants. This number is in keeping with the widely accepted sample size of 30–50 participants for pilot studies [15-17]. On a practical level, we aimed to recruit enough participants to fill 4 classes (2 IA and 2 DAC groups of 12). This sample was expected to be sufficient to identify any problems of recruitment, delivery of the intervention and evaluation measures.

Multiple recruitment methods were employed in the study. Potential participants were recruited via the websites, phone lines, newsletters and waiting lists from two national charities for depression (i) the Scotland based charity, Action on Depression, and (ii) AWARE Defeat Depression in Derry/Londonderry, Northern Ireland. This strategy was supplemented by advertisements in local newspapers (e.g. Metro) and posters in community locations such as cafes. No participants were recruited directly from the UK National Health Service (NHS).

Individuals were included in the study if they were 16 years old or more, had at least mild depressive symptoms identified by a minimum score of 5 on the Patient Health Questionnaire (PHQ-9) [18], and gave consent to take part. Participants were excluded if they could not read, speak and understand English, if they did not consent to abide by normal social etiquette within the classes and/or if their score on the PHQ-9 was below 5. As the study was a pilot study, we felt that we should not exclude individuals based on changes in antidepressant medication or participation in psychotherapeutic intervention, therefore, these variables were not included in the exclusion criteria but were recorded to better describe the sample.

### Procedure and randomisation

Baseline measures were collected from eligible participants two weeks prior to the beginning of the first course. Participants were then remotely randomised to either the IA or DAC group. Randomisation was carried out by the Robertson Centre for Biostatistics at the University of Glasgow, part of the Glasgow Clinical Trials Unit, by staff not in direct contact with participants, and who did not carry out the final analysis. As part of the randomisation process, participants were stratified based on three criteria 1. Age: Older than 40/Younger than 40, 2. Gender: Male/Female, 3. Use of antidepressant medication: Taking antidepressants/Not taking anti-depressants, in order to aid equal distribution of these factors across the two groups.

Following randomisation, participants were informed of the group they had been assigned to (IA or DAC) and told the date and time of their first class. The IA group attended the classes 2 weeks post-baseline and the DAC group’s course started approximately 14 weeks post-baseline.

### Intervention

The ‘Living Life to the Full’ classes are based on the principles of CBT and teach life skills, covering various topics relating to depression and anxiety [19]. Participants were invited to attend eight 90-minute weekly classes which were held across the two sites (Glasgow and Derry/Londonderry) in community buildings such as libraries. Each class focused on a different common problem faced by people when they feel low or anxious. The course content included the following topics: Week 1- Why do I feel so bad?; Week 2- I can't be bothered doing anything; Week 3- Why does everything always go wrong?; Week 4- I'm not good enough; Week 5- How to fix almost everything; Week 6- The things you do that mess you up; Week 7- Are you strong enough to keep your temper?; and Week 8–10 things you can do to feel happier straight away. A single revision session called “Planning for the future” was also held 6-weeks after the final session.

Classes were led by two experienced and trained self-help coaches provided by the two charities. All trainers attended a training workshop on delivering the classes. Sessions were manualised/scripted by the authors of the intervention and trainers used a course CD which contained speaker notes and un-editable slides which were presented to participants during the sessions. Participants were provided with an accompanying course book at each session. These served as the CBT self-help material, guided and supported by class participation.

Participants carried out various individual and group tasks at each session in order to understand the content, apply it to themselves and to practice some of the techniques that had been introduced. Participants were encouraged to work through the accompanying session book in their own time and complete structured homework tasks using worksheets such as the ‘plan, do, review’ model in which participants formulate a clear, manageable plan for applying a skill or piece of advice that they have learned. Expected obstacles were identified and the plans adapted accordingly. The following week a review sheet was used to self-assess the success of participants’ plans [20].

### Outcome measures

The aim of the study was to test recruitment, retention, acceptability and ability to gather data, as well as inform the power calculation for the future substantive RCT. Following baseline assessments, all follow-up measures were recorded at both 3 and 6 months to estimate the change in depression (PHQ-9) [18], and anxiety (Generalised Anxiety Disorder-7 [GAD-7]) [21]. Participant satisfaction was measured in the IA group using the Client Satisfaction Questionnaire (CSQ-8) [22] and by describing attendance at the classes and feedback on each session. These outcome measures are widely used in this area of research and are outlined below.

#### PHQ-9

The PHQ-9 is a freely available mood rating questionnaire consisting of nine questions mirroring DSM-IV depression diagnostic criteria [18]. The PHQ-9 has shown diagnostic validity in a study of 3,000 adult patients. Each item is rated on a scale of 0 to 3, giving a maximum score of 27. Cut-off scores are used to label depression severity as: 0 to 4, minimal depression; 5 to 9, mild depression; 10 to 14, moderate depression; 15 to 19, moderately severe depression; 20 to 27, severe depression.

#### GAD-7

The GAD-7 [21] is a seven-item questionnaire focusing on symptoms of anxiety experienced in the past 2 weeks. Each item is rated according to the frequency of the described problem. The responses are scored as follows: 0 = ‘not at all’, 1 = ‘several days’, 2 = ‘more than half the days’, 3 = nearly every day’ with a maximum score of 21 Scores are interpreted as 5 to 9, mild anxiety; 10 to 14, moderate anxiety; and 15 and above, severe anxiety. The GAD-7 showed good reliability and criterion, construct, factorial, and procedural validity in a study carried out in 15 primary care clinics [21].

#### CSQ-8

The client satisfaction questionnaire (CSQ-8) was administered post-intervention as a measure of satisfaction with the intervention. The CSQ-8 is an eight-item questionnaire rated using a four-point Likert scale. Scores range from 8 to 32 with higher scores indicating greater satisfaction with the intervention in question. A series of studies of this measure have indicated high internal consistency (Cronbach’s alpha 0.92 to 0.93) as well as criterion and construct validity [22].

Furthermore, participants were invited to complete a class feedback form at the end of each session. Participants were asked to provide insight regarding what was helpful or not helpful about each session. Participants were also invited to participate in a short interview with the research assistant following completion of the 8-week course. The aim of these interviews was to gain further insight into the delivery of the LLTTF classes and useful information that could help improve future delivery of the sessions. This results of these interviews will be published separately.

### Statistical methods

Participant uptake and adherence to classes were summarised and presented as percentages. Qualitative data gained from session feedback forms were analysed for useful insight regarding the delivery and content of the classes. Baseline characteristics were summarised for all participants as well as for the IA and DAC groups separately. Two-sample t-tests and Fisher’s exact tests were used to compare the two treatment groups and confirm that participants had been effectively randomised.

To assess efficacy, repeated measures analysis was carried out using an intention to treat approach by means of a linear mixed effects model with time (baseline, 3 months, 6 months) , group (control, intervention) and their interaction as fixed effects, and participant as a random effect. Models were adjusted for age, gender and antidepressant use at baseline. Tukey post-hoc comparisons were carried out with a familywise error rate of 5%. These models were used to estimate, and test the statistical significance of the within-group differences between time points and the between-group differences at each time point and the estimate of clinical effect and drop-out/non data retention rates used to provide a power calculation needed for the future substantive study.

## Results

### Recruitment

**Figure 1:**
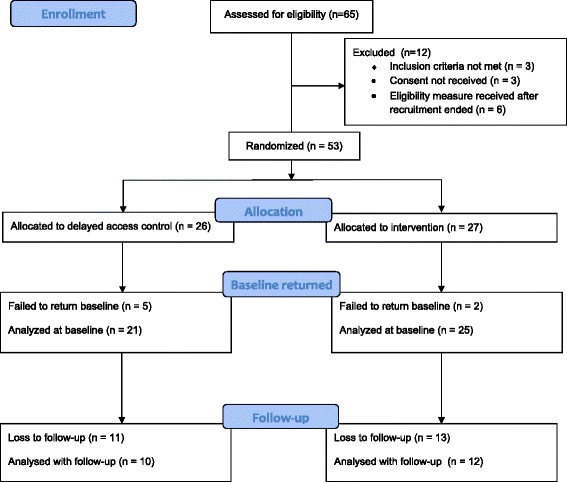
**CONSORT diagram.** Flow diagram for the study outlining the enrolment, screening, allocation and follow-up of participants.

Sixty-five individuals expressed an interest in participating, of which 12 were excluded. Fifty-three participants were recruited (29 in Scotland and 24 in Northern Ireland) in a combined recruitment period of approximately 6 weeks. Of these, there were no baseline PHQ-9 data recorded for 7 patients who were excluded from analysis. The final analysis is carried out on data from the 46 participants that were randomised and provided baseline measures (27 in Scotland and 19 in Northern Ireland) (Figure 1).

Demographics and baseline characteristics are outlined below in Table 1. There were no statistically significant differences between study groups at baseline.

**Table 1:** **Characteristics of participants at baseline**

***Characteristic***	***Statistic***	***Overall n = 46***	***Control n = 21***	***Intervention n = 25***	P-value*
**Age**	mean (sd)	43.7 (13.0)	44.8 (14.6)	42.6 (11.0)	0.589
**Sex: Male**	n (%)	16 (35%)	9 (43%)	7 (20%)	0.360
**Medication: Yes**	n (%)	24 (52%)	11 (52%)	13 (52%)	1.000
**Site: Northern Ireland**	n (%)	19 (41%)	8 (38%)	11 (44%)	0.769
**Chronicity**					0.224
**<1 year**	n (%)	1 (2%)	0 (0%)	1 (4%)	
**>1 year**	n (%)	43 (93.5%)	20 (95.2%)	23 (92%)	
**Not all the time**	n (%)	1 (2%)	0 (0%)	1 (4%)	
**Not given**	n (%)	1 (2%)	1 (5%)	0 (0%)	
**PHQ-9**	mean (sd)	14.5 (6.5)	14.3 (6.9)	14.7 (6.4)	
**GAD-7**	mean (sd)	12.0 (5.5)	12.8 (6.1)	11.3 (4.9)	

*P-values from two-sample t-tests and Fisher’s tests, for continuous and categorical variables, respectively.

### Help-seeking

Upon entry into the study, participants were asked to indicate any support they were currently receiving for their low mood. Participants could select all relevant sources of support. The most common form of support currently being received was from participants’ GP (54.3%), followed by help from family/friends (45.7%) and self help (37%). Fewer than 25% of participants were receiving talking therapy or counselling upon entry into the study and 13% were receiving no support.

### Adherence - class attendance

**Figure 2:**
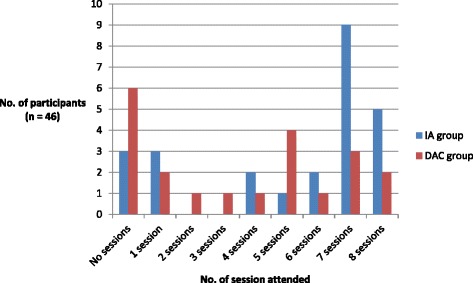
**Adherence to the intervention.** Class attendance in both the IA and DAC groups – the number of participants that attended each of the 8 sessions.

Attendance was recorded for both IA and DAC participants (n = 46) (see Figure 2). Participants attended on average 4.46 (sd = 3.06) of the 8 course sessions. Overall 80.4% of participants attended at least one session, 65.2% attended half of the sessions, 47.8% attended 6 or more and 15.2% attended all of the 8 sessions.

### Participant satisfaction

Participant satisfaction with the LLTTF classes was measured using the CSQ-8 for the intervention group. A mean satisfaction score of 28 (sd = 4.8), from a possible score of 32, indicated that participants were highly satisfied with the class content and delivery (n = 27). Written comments from class feedback forms provided useful information regarding delivery of the LLTTF classes. Participants identified several factors which they found particularly helpful including: sharing experiences with peers within the group setting; action planning; the simplicity and usability of the self-help books; learning positive affirmations; reviewing past goals; and being guided by motivated class leaders.

Minimal but useful information was also obtained on factors which were not helpful to the sessions. These included issues to do with the environment (“The room was too hot”), method of delivery (“Don’t like group feedback to class”), or class content (“Thinking about bad things I say about myself”).

### Ability to collect data/questionnaires

At 3 months follow-up, data was collected from 74% of participants. This dropped to 48% at 6 months. The amount of missing data was similar across the two groups: 29% and 24% for DAC and IA respectively at 3 months and 52% in both groups at 6 months. Fisher’s exact tests show no evidence of a difference in loss to follow-up in either group at either follow-up time (3 months: p =0.988; 6 months: p = 1.00).

### Initial assessments of efficacy

Within-group differences from baseline to both follow-ups are shown for both measures in Table 2. Note that, in both instruments, a decreased score is indicative of improvement.

**Table 2:** **Within-group differences estimated from linear mixed effects models, adjusted for age, gender and medication use, from baseline to 3 month follow-up and baseline to 6 month follow-up, for both depression (PHQ-9) and anxiety (GAD-7) scores**

***Response***	***Follow-up***	***Group***	***Difference from baseline***		
			***Estimate***	***95% CI***	***P-value***
**PHQ-9**	3 months	DAC	−2.14	(−5.95, 1.66)	0.583
		IA	−7.49	(−10.90, −4.08)	<0.001
	6 months	DAC	−6.57	(−11.02, −2.11)	<0.001
		IA	−8.30	(−12.57, −4.44)	<0.001
**GAD-7**	3 months	DAC	−0.48	(−4.65, 3.68)	0.999
		IA	−6.24	(−9.97, −2.50)	<0.001
	6 months	DAC	−6.14	(−10.98, −1.30)	0.004
		IA	−6.37	(−10.79, −1.96)	<0.001

#### Effect on depression (PHQ-9) scores

While the average between-group difference in PHQ-9 score at 3 months, adjusted for age, gender and medication use, was suggestive of an improvement in the immediate access group, (mean difference: 5.25 units), as expected in this pilot study this was not statistically significant at the 5% level (p = 0.106, 95% CI (−0.59, 11.09)).

#### Effect on anxiety (GAD-7) scores

At 3 months, the between-group difference in anxiety scores, as measured by the GAD-7,was on average, 6.98 units lower than the control group (p = 0.001, 95% CI (1.87, 12.09)). No between-group difference was found at the 6 month follow-up (mean difference = −1.46 units, 95% CI (−4.65, 7.58), p = 0.984).

## Discussion

This pilot study aimed to test key elements of a proposed future substantive RCT of the Living Life to the Full classes recruited from the community and delivered in the voluntary sector. It demonstrated that a target group of self-referring participants can be successfully recruited from the community and engaged in the RCT. Several key findings were identified.

### Recruitment and sample

Firstly, recruitment was feasible with 46 eligible individuals recruited within a short 6-week timeline and successfully randomised to an immediate access or delayed access group. Recruitment methods which focussed on community promotion effectively reached individuals with moderate to moderately severe depression (mean PHQ-9 score 14.5) and moderate levels of anxiety (mean GAD-7 score 12.0). A high proportion of individuals reported having symptoms of depression and/or anxiety for greater than 2 years (n = 37, 72%) and 57% over five years reflecting a chronic group. More than half of the participants (n = 24, 52%) were taking prescribed anti-depressant medication indicating they had previously presented to the health service for management of their symptoms. Overall 45.7% were not being currently seen by their GP. This is in line with research showing that only half of individuals with depression seek help from the health service [11]. Our community-based approach, therefore, has the potential to address this treatment gap by reaching individuals who would not otherwise engage with the health service.

### Intervention delivery and satisfaction

We have shown it is feasible to deliver the intervention classes in a community setting via the voluntary sector. Attendance and adherence to the LLTTF classes was reasonable, with participants attending on average 50% of the 8-week course. Participant satisfaction (CSQ-8 score of 28 (sd = 4.8)) was higher than group interventions delivered in either primary care (25.3 (sd = 5.8)) [23] or outpatient settings (24.4 (sd = 3.5)) [24]. Written feedback from participants identified minimal negative aspects, helping to explain the reasonable levels of attendance and high satisfaction. Positive feedback was mainly associated with the group setting, the accessibility of the resources, and useful behaviour change techniques used by the class leaders. In particular, group-based education has been shown to facilitate behaviour change by providing peer-led motivation and support [25].

### Data collection and follow-up

Secondly, it proved possible to record data, with a 74% follow-up rate at 3 months, although this was lower (48%) at 6 months. This indicates the need to introduce additional strategies to retain participants at longer-term follow-up, possibly by the use of incentives.

### Clinical effect

Although the pilot study was not intended to answer the question of efficacy, at the 3 month follow-up, a significant improvement was found between the intervention and control group for levels of anxiety. Although not statistically significant, an improvement was also observed in levels of depression. The PHQ-9 data was used for a power calculation for the full RCT.

### Power calculation

For the future study we wish to power the calculation targeted at a group who have higher scores for depression, as bibliotherapy for depression is known to be more effective for participant groups who start off with a score of at least moderate depression (a score of 10 or more on the PHQ9) at baseline [23]. Data collected from the current pilot RCT, from a mixture of intervention and control group participants, showed that for those with a PHQ-9 score of 10 or more at baseline, mean PHQ-9 scores reduced from 17.7 to 10.8 points after the intervention, with a standard deviation of the changes of 6.1 points. This is a group of people who are most likely to benefit from the classes, and also who might potentially be accepted for treatment by health care practitioners. We would intend however to use the same recruitment strategy so that we seek to include people scoring 5 or more on the PHQ9, thereby ensuring that groups fill up to sufficient numbers hereby preventing delay. The primary analysis in the future substantive RCT will, compare changes in PHQ-9 scores in those scoring 10 or more on the PHQ9 at 6 months between intervention groups in all participants. We will power the study to be able to detect a between-group difference of 5.5 points on the PHQ-9 score. A difference of 5.5 points is used to reflect a category change on the PHQ-9 and is clinically important.

Based on a two-sample *t*-test, a sample size of 27 participants per arm would be required to have 90% power. In the pilot, follow-up data was available for 74% at 3 months and for 48% at 6 months. The proposed study would have one follow-up assessment at 6 months, so we expect follow-up rates between these two values. To allow for a 65% follow-up rate 84 people (two groups of 42) with PHQ-9 scores of 10 or more would need to be randomised. In this pilot, one-third of participants had PHQ-9 scores less than 10 points at baseline, so that 126 participants in total (two groups of 63) would need to be randomised in the future large RCT. We would also need to ensure that the required number of participants scoring more than 10 on the PHQ-9 have entered the study before ending recruitment.

### Limitations

This was a small pilot study, designed to test whether such a trial is viable, has been very valuable in calculating the required sample size for the future RCT and ascertaining that such a trial is feasible.

We did not determine whether the participants had a formal diagnosis of depression or anxiety, rather, participants were permitted entry into the study based on self-reported levels of low mood. To improve this aspect of the research design, the future RCT will include a diagnostic interview. Eligible participants will be invited to take part in a telephone call to confirm their diagnosis using the Mini International Neuropsychiatric Interview **(**MINI) [26]. We anticipate that most, if not all, participants will agree to the interview. However, for some people an attraction of the voluntary sector delivery is that they can seek help without creating a formal NHS record. Some potential participants may, therefore, rather not take part in a diagnostic interview, so individuals who enter the study will be given the option to decline the interview, if they prefer. The MINI will be useful in describing the population being studied but will not be used as an outcome measure.

### Future research

A decision was made to also extend the DAC to 6 months in the future study to extend the length of follow-up. This means that there will be a control group, who has not received the intervention, at this longer term follow-up point. Additionally, due to the possible financial benefits of low intensity interventions and the stepped care approach [27], an economic analysis will be included in the future substantive RCT. Additionally, it may be beneficial for future studies to investigate the efficacy of this intervention in socially isolated groups such as older adults, a group which may be more difficult to engage and treat in this way [28].

## Conclusions

Our results show that it is likely to be feasible to conduct a substantive RCT within a community-based setting, delivered by individuals from the voluntary sector. The power calculation suggests a sample size of 126 is required. This pilot study strongly supports our potential to recruit this required number of participants using our multi-point recruitment strategy. Although the study was not powered to find such a result, we found that the LLTTF classes appeared to improve symptoms of anxiety. Delivery of the intervention was associated with high participant satisfaction and reasonable attendance. These findings include a sample size calculation to inform the future larger scale RCT, with the aim being to further explore the effectiveness of the Living Life to the Full classes in the management of depression and anxiety in a community setting.

### Ethical approval

Ethics approval was granted by the College of Medical, Veterinary & Life Sciences Ethics Committee for Non Clinical Research Involving Human Subjects, University of Glasgow- (Reference FM01910).

## Abbreviations

CBT: Cognitive behavioural therapy
RCT: Randomised controlled trial
NICE: National institute for health and care excellence
NHS: National health service
LLTTF: Living life to the full
IA: Immediate access
DAC: Delayed access control
